# Fracture risk prediction, treatment thresholds, and the role of bone mineral density: a comparative analysis of FRAX and SHAFRE

**DOI:** 10.1007/s00774-025-01684-x

**Published:** 2026-01-03

**Authors:** Anna Nordström, Marcel Ballin, Viktor Ahlqvist, Peter Nordström

**Affiliations:** 1 Department of Medical Sciences, Rehabilitation Medicine, Uppsala University, Uppsala, Sweden; 2 School of Sport Sciences, UiT The Arctic University of Norway, Tromsø, Norway; 3 Department of Public Health and Caring Sciences, Clinical Geriatrics, Uppsala University, Uppsala, Sweden; 4 Department of Biomedicine, Aarhus University, Aarhus, Denmark; 5 Institute of Environmental Medicine, Karolinska Institutet, Stockholm, Sweden

**Keywords:** Prediction, Fractures, FRAX, SHAFRE

## Abstract

**Introduction:**

Accurate prediction of fracture risk is important for treatment decisions, yet evaluations of commonly used thresholds are limited, and the added value of volumetric bone mineral density (vBMD) remains uncertain. We conducted the first head-to-head comparison of the SHAFRE and FRAX algorithms using identical predictor information.

**Materials and methods:**

We included 3525 community-dwelling Swedish men and women (mean age 71.4 years) who underwent a health examination with femoral neck areal BMD (aBMD) and radial vBMD. Incident fractures were retrieved from the National Patient Register. Model performance was evaluated using threshold-specific sensitivity and specificity.

**Results:**

Over a mean follow-up of 8.7 years, 559 participants sustained a fracture. SHAFRE predicted a mean 10-year fracture risk of 21.9% in those who fractured versus 15.6% in the remaining cohort (ROC-area: 68%, 95% CI: 66–71%). Corresponding FRAX values were 16.7% and 12.6% (ROC-area: 66%, 95% CI: 63–68%), and FRAX slightly underestimated fracture risk in this cohort. Adding radial vBMD on top of aBMD did not materially improve discrimination for SHAFRE (70%, 95% CI: 67–72%) or FRAX (68%, 95% CI: 65–70%). Threshold-specific analysis identified an optimal predicted risk threshold of 13–17%, consistently below the commonly recommended 20%.

**Conclusions:**

Predictive ability for major fractures remained modest for both algorithms and was not improved by adding vBMD. The estimated optimal threshold for treatment initiation was lower than the commonly recommended 20%. These findings reinforce that discrimination is substantially stronger for hip fracture than for major osteoporotic fractures.

## Introduction

Bone fractures in older adults pose a major public health burden, leading to significant healthcare costs, morbidity, and loss of independence. Among people aged 50 years and over, one in three women and one in five men will sustain an osteoporotic fracture in their lifetime [1–4]. Despite advances in prevention, fracture burden continues to rise, with 178 million new cases reported in 2019 alone, a 33% increase since 1990 [5]. This underscores the importance of precise fracture risk prediction models and well-validated treatment thresholds.

In osteoporosis management, clinicians are facing a major challenge in terms of having to decide *when* to initiate bone-specific treatment. Current guidelines frequently adopt a 20% 10-year risk threshold for major osteoporotic fractures, based on estimates from fracture algorithms, such as FRAX [6]. However, the scientific justification for this threshold remains unclear as few studies have systematically evaluated its validity. A review of 120 international osteoporosis guidelines found that nearly all rely on the National Osteoporosis Foundations 20% threshold, with minimal independent assessment of its appropriateness for different populations [7]. This raises concerns about whether current thresholds truly reflect an optimal risk–benefit balance for treatment initiation.

Beyond treatment thresholds, the accuracy of fracture prediction models remains a key issue [8]. Few studies have compared different fracture algorithms or assessed whether incorporating volumetric bone mineral density (vBMD) enhances predictive accuracy beyond traditional predictors. Therefore, this study aims to compare the predictive performance of SHAFRE [9] and FRAX, examines the impact of including both areal BMD (aBMD) and vBMD, and reassesses whether the widely used 20% threshold remains appropriate.

## Materials and Methods

### Study design and setting

This study was conducted within the Healthy Aging Initiative (HAI), a primary prevention study initiated in Umeå, Sweden (2012–2022). The HAI aimed to investigate the prevalence of risk factors for non-communicable diseases, with a particular focus on fractures, diabetes, and cardiovascular disease among 70-year-old men and women [10]. Participants were identified through national population registers and invited via mailed information followed by a telephone call upon reaching 70 years of age. Those who consented provided written informed consent and underwent a comprehensive health assessment at a dedicated research clinic. During 2012–2019, about 55% of the 70-year-old inhabitants of Umeå municipality participated in HAI. Of 4,971 individuals who underwent aBMD assessment of the proximal femur (2012–2019), 3525 also had vBMD of the distal radius measured and were included in this study. This study was designed and reported in accordance with the TRIPOD (Transparent Reporting of a multivariable prediction model for Individual Prognosis Or Diagnosis) guidelines.

### Predictors

Predictors were selected based on established fracture risk predictors used in SHAFRE and FRAX [6, 9]. For SHAFRE, these included areal BMD (aBMD, g/cm^2^), age, sex, whether the individual was born in the Nordic countries, hip fracture in sibling, use of home care service, and all diagnoses and medications listed in Table 1. The predictor nursing home resident was excluded since only three individuals lived in nursing homes at baseline. For FRAX, these included aBMD, age, sex, body weight, height, smoking status, use of prednisolone, diagnoses of previous fractures, rheumatoid arthritis, a diagnosis of osteoporosis, and alcohol abuse. Because we did not have data on hip fracture in parent (as originally used in FRAX), we instead included hip fracture in sibling. Diagnoses were obtained from the Swedish National Patient Register [11] (covering all inpatient and outpatient specialized care) using ICD-10 codes (Table 1) and medications were obtained from the Prescribed Drug Register (covering all dispensed prescription medications in Sweden) using appropriate Anatomical Therapeutic Chemical Classification System codes (Table 1). Siblings with hip fractures were identified using linked data from Statistics Sweden [12] and the National Patient Register. Areal BMD (g/cm^2^) of the femoral neck, total proximal femur, and lumbar spine (L1–L4) was measured using Lunar I-DEXA (GE Healthcare). DXA uses low-dose X-rays at two energy levels to differentiate between bone and soft tissue, providing precise aBMD values (g/cm^2^) and a coefficient of variation of about 1% [13]. For the present study, we present data for femoral neck aBMD only as this measure is used in the FRAX algorithm. The equipment was calibrated daily according to manufacturer recommendations. Volumetric BMD (vBMD, mg/cm^3^) was assessed using a Stratec XCT 2000 (Stratec Medizintechnik, Germany). This technique provides three-dimensional assessments of bone geometry and vBMD (mg/cm^3^). Scans were performed at the 4% distal radius, primarily capturing trabecular and cortical bone parameters. Measurement reproducibility has been validated, with coefficients of variation typically below 1% [14]. For this study, total vBMD at the 4% distal radius site was used. Data were complete for all covariates, except for smoking status, that was missing for six individuals.

**Table 1:** Descriptive characteristics of the total cohort (*N* = 3525) at baseline, and based on fracture during follow-up

	Total cohort	Fracture during follow-up		*p*-value
	*N* = 3525	Yes, *N* = 559	No, *N* = 2966	
*Variable*				
Age, yrs ± SD	71.4 ± 2.0	71.3 ± 2.0	71.4 ± 2.0	0.64
Weight, kg ± SD	76.8 ± 14.7	74.3 ± 14.3	77.3 ± 14.7	< 0.001
Height, cm ± SD	170 ± 9	168 ± 9	170 ± 9	< 0.001
Female sex	1778 (50.4%)	371 (66.4%)	1407 (47.4%)	< 0.001
Born in the Nordic countries	3,498 (99.2%)	557 (99.6%)	2941 (99.2%)	0.37
Sibling with hip fracture	100 (2.8%)	13 (2.3%)	87 (2.9%)	0.90
Home care service	27 (0.8%)	8 (1.4%)	19 (0.6%)	0.02
Smoking	205 (5.8%)	43 (7.7%)	162 (5.5%)	0.06
Diagnoses, ICD-code*, N (%)				
Alcohol abuse, F10	38 (1.1%)	10 (1.8%)	28 (0.9%)	0.16
Chronic pulmonary disease, J44	48 (1.4%)	5 (0.9%)	43 (1.4%)	0.69
Dementia, F00, F01, F039, G30	19 (0.5%)	5 (0.9%)	14 (0.5%)	0.94
Osteoporosis, M81	49 (1.4%)	16 (2.9%)	33 (1.1%)	0.01
Parkinson's disease, G20	29 (0.8%)	9 (1.6%)	20 (0.7%)	< 0.001
Fracture, S12–S82, excluding S62	608 (17.2%)	150 (26.8%)	458 (15.4%)	< 0.001
Renal disease, N17–N19	30 (0.9%)	10 (1.8%)	20 (0.7%)	0.57
Rheumatoid disease, M05	80 (2.3%)	22 (3.9%)	58 (2.0%)	0.34
Stroke, I61, I63, I64	124 (3.5%)	28 (5.0%)	96 (3.2%)	0.05
*Medications, ATC-code*^*#*^*, N (%)*				
Antidepressants, N06A	759 (21.5%)	157 (28.1%)	602 (20.3%)	< 0.001
Prednisolone, H02AB06	728 (20.7%)	132 (23.6%)	596 (20.1%)	0.50
Neuroleptics, N05A	82 (2.3%)	18 (3.2%)	64 (2.2%)	0.21
*Bone density measurements*				
Femoral neck aBMD, g/cm^2^ ± SD	0.87 ± 0.14	0.81 ± 0.12	0.88 ± 0.14	< 0.001
Radius vBMD, mg/cm^3^ ± SD	286 ± 60	259 ± 56	291 ± 59	< 0.001

^*^International classification of disease, version 10

^#^Anatomical therapeutic chemical classification

### Outcome measurement

The primary outcome was time to first incident fracture after baseline, identified in the NPR and classified using ICD-10 codes (S12, S22, S32, S42, S52, S72, and S82). A fracture was considered incident if:

- It was the first recorded fracture for the individual, occurring after baseline.
- It occurred after baseline and ≥ 12 months after a prior recorded fracture, to avoid double-counting the same clinical event.

The positive predictive value of fracture diagnoses in the NPR ranges from 70 to 87%, with the highest accuracy for hip fractures [15–18]. Deaths during follow-up were captured via the Swedish Cause of Death Register [19] and used for censoring in survival analysis. Follow-up continued for up to 10 years post-baseline.

### Ethical approval

This research strictly adheres to the guidelines outlined in the World Medical Association's Declaration of Helsinki and has obtained ethical approval from both the local Ethics Committee and subsequently the Swedish Ethical Review Authority (Number 07–031 with extensions). Prior to their involvement, all participants provided written informed consent to participate in the study.

### Statistical analysis

Descriptive statistics were presented as means ± standard deviations (SD) for continuous variables and as counts (percentages) for categorical variables.

#### Fracture risk estimation and model performance

To assess the independent effects of predictors (Table 1), flexible parametric survival models were used to estimate the hazard ratio and failure (1-Survival) of fracture over time, modeling the log cumulative hazard function with restricted cubic splines (three degrees of freedom, and default knot positions) [20]. All hazards models were multivariable, including all predictors simultaneously.

The date of bone density measurement was defined as the start of follow-up for the present study. The primary endpoint was time to first fracture, but individuals were censored also at date of death, or last follow-up (31 November 2024), whichever occurred first. Individual fracture risk over 10 years was estimated using:

- SHAFRE: Baseline hazard function and each individual’s covariate profile (https://www.healthy-ageing.life/shafre-fracture-risk-assessment)
- FRAX: Swedish FRAX model (https://frax.shef.ac.uk/FRAX/tool.aspx?country=5)

#### Model performance evaluation

The discriminative ability of each model was assessed using the area under the receiver operating characteristic curve (ROC), computed with the ROCTAB command in Stata.

Three models were evaluated for SHAFRE and FRAX respectively:

1. Baseline model: without bone density.
2. BMD-enhanced model: including femoral neck aBMD.
3. Comprehensive model: including femoral neck aBMD and radius vBMD.

#### Threshold optimization for treatment initiation

To determine a potential optimal fracture risk threshold for initiating bone-specific treatment, we estimated sensitivity, specificity, and ROC-area for different risk thresholds in both the SHAFRE and FRAX models.

#### Model calibration and agreement

Concordance plots were used to visually assess agreement between predicted and observed fracture risks. A scatter plot displayed individual data points, with a 45-degree reference line (red, dashed) indicating perfect concordance. A LOESS regression curve (black, solid) with a 95% confidence interval was overlaid to illustrate the relationship between predicted and observed risks.

All analyses were performed using SPSS (version 28) and Stata (version 16.1 MP). A two-sided p-value < 0.05 or 95% confidence intervals (CIs) excluding one were considered statistically significant.

## Results

### Baseline characteristics and fracture incidence

This study included 3525 men and women with a mean age of 71.4 years at baseline (Table 1). The most common baseline diagnosis included previous fractures (17.2% in the cohort at baseline), while antidepressants were the most frequently prescribed medication (21.5%) at baseline. Additionally, 5.8% of participants were smokers.

Over a mean follow-up of 8.7 years (standard deviation [SD] 2.6 years, range 0–10 years), 559 participants (15.9%) sustained a fracture. These included 160 forearm fractures, 87 upper arm and shoulder fractures, 85 lower leg and ankle fractures, 90 femoral fractures, and 137 spine, rib, or pelvic fractures. Participants who fractured were shorter, weighed less, were more often female, had a history of fractures or Parkinson’s disease, and were more frequently prescribed antidepressants (Table 1). Femoral neck aBMD and vBMD of the distal radius were also lower in those who fractured.

### Independent predictors for fracture

The independent association between each predictor and incident fracture is shown in Table 2. The strongest predictors (p < 0.01 for all), included higher body height (Hazard ratio [HR], 1.02, 95% CI, 1.01–1.04 per 1 cm increase), female sex (HR, 1.59, 95% CI, 1.20–2.12), a previous fracture (HR, 1.45, 95% 1.19–1.76), renal disease (HR, 2.72, 95% CI, 1.44–5.13), stroke (HR, 1.79, 95% CI, 1.21–2.63), femoral neck aBMD (HR, 0.73, 95% CI, 0.66–0.82 per SD increase), and radius vBMD (HR, 0.73, 95% CI, 0.65–0.82 per SD increase).

**Table 2:** Independent risk factors for fracture (*N* = 559) during follow-up in the cohort (*N* = 3525) were investigated using Cox regression

Variable	Hazard ratio	95% CI	*p*-value
Age, per yr	1.00	0.95, 1.04	0.90
Weight, per kg	1.00	1.00, 1.01	0.42
Height, per cm	1.02	1.01, 1.04	0.001
Female sex	1.59	1.20, 2.12	0.001
Born in the Nordic countries	2.05	0.51, 8.25	0.31
Sibling with hip fracture	0.67	0.39, 1.17	0.16
Home care service	1.54	0.73, 3.24	0.26
Smoking	1.26	0.92, 1.73	0.16
*Diagnoses, N (%)*			
Alcohol abuse	1.74	0.92, 3.31	0.09
Chronic pulmonary disease	0.49	0.20, 1.20	0.12
Dementia	1.71	0.69, 4.20	0.25
Osteoporosis	1.29	0.77, 2.16	0.33
Parkinson's disease	2.25	1.15, 4.41	0.02
Fracture	1.45	1.19, 1.76	< 0.001
Renal disease	2.72	1.44, 5.13	0.002
Rheumatoid disease	1.39	0.89, 2.17	0.15
Stroke	1.79	1.21, 2.63	0.003
*Medications, N (%)*			
Antidepressants	1.26	1.04, 1.53	0.02
Prednisolone	1.15	0.94, 1.41	0.19
Neuroleptics	1.11	0.66, 1.85	0.70
*Bone density measurements*			
Femoral neck aBMD, per SD	0.73	0.66, 0.82	< 0.001
Radius vBMD, per SD	0.73	0.65, 0.82	< 0.001

Hazard ratios (HR) and 95% confidence intervals (95% CI) are presented

### Fracture prediction using SHAFRE

**Fig. 1:**
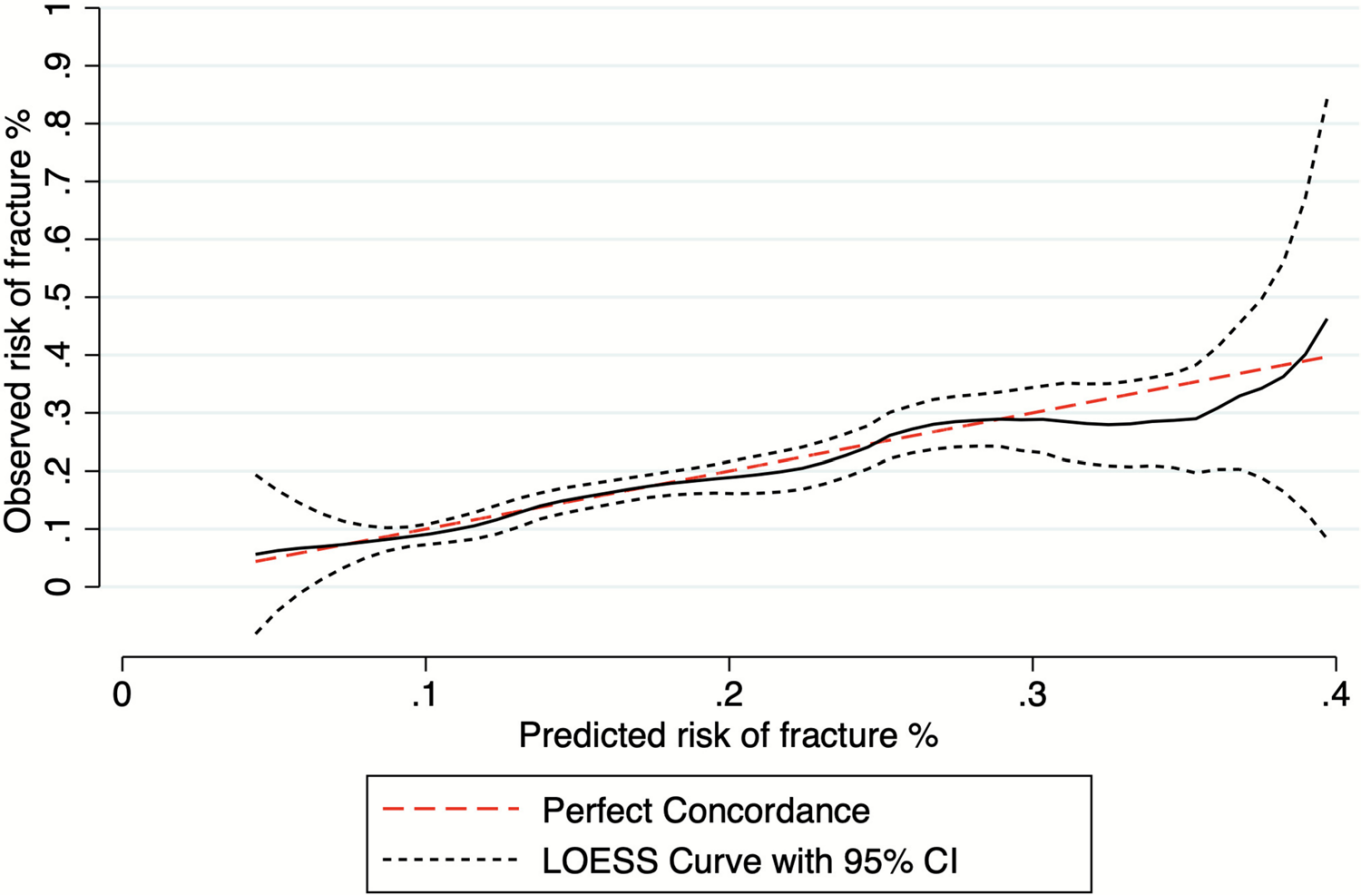
Concordance plot illustrating the predicted risk of fractures compared to the observed risk including all predictors used in SHAFRE

**Fig. 2:**
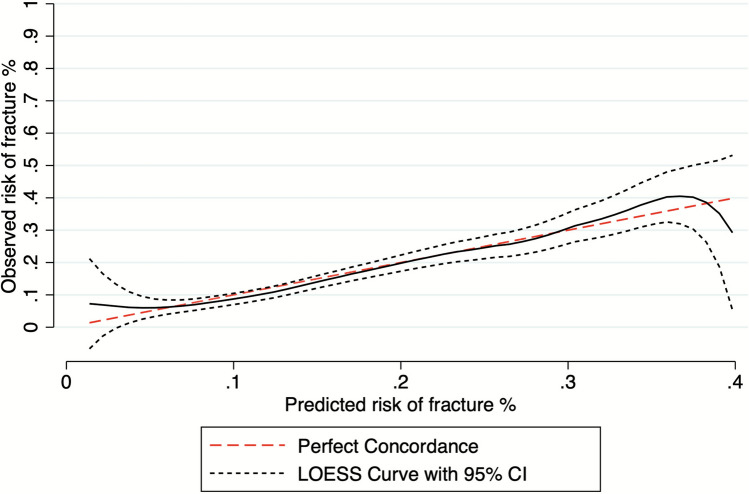
Concordance plot illustrating the predicted risk of fractures compared to the observed risk including all predictors used in SHAFRE and neck aBMD

**Fig. 3:**
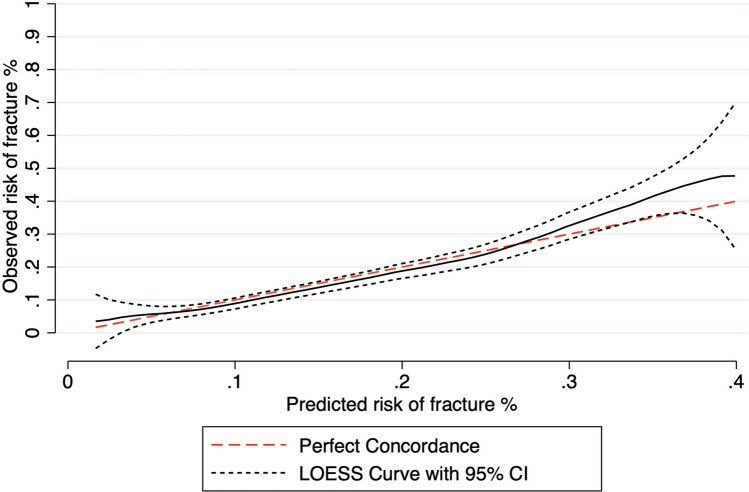
Concordance plot illustrating the predicted risk of fractures compared to the observed risk including all predictors used in SHAFRE, neck aBMD and radius vBMD

Using the SHAFRE algorithm without bone density, the mean predicted 10-year fracture risk was 20.4% in those who fractured and 15.9% in the rest of the cohort). The ROC-area was 64% (95% confidence interval 62–67%). A concordance plot suggested good accuracy of the model (Fig. 1). Adding femoral neck aBMD changed the predicted risk to 21.9% in those who fractured and 15.6% in those without fracture, with a ROC-area of 68% (95% CI, 66–71%, Fig. 2). Including both femoral neck aBMD and radius vBMD slightly increased the predicted risk: 22.6% and 15.6% respectively, and a ROC-area of 70% (95% CI, 67–72% (Fig. 3).

### Fracture prediction using FRAX

**Fig. 4:**
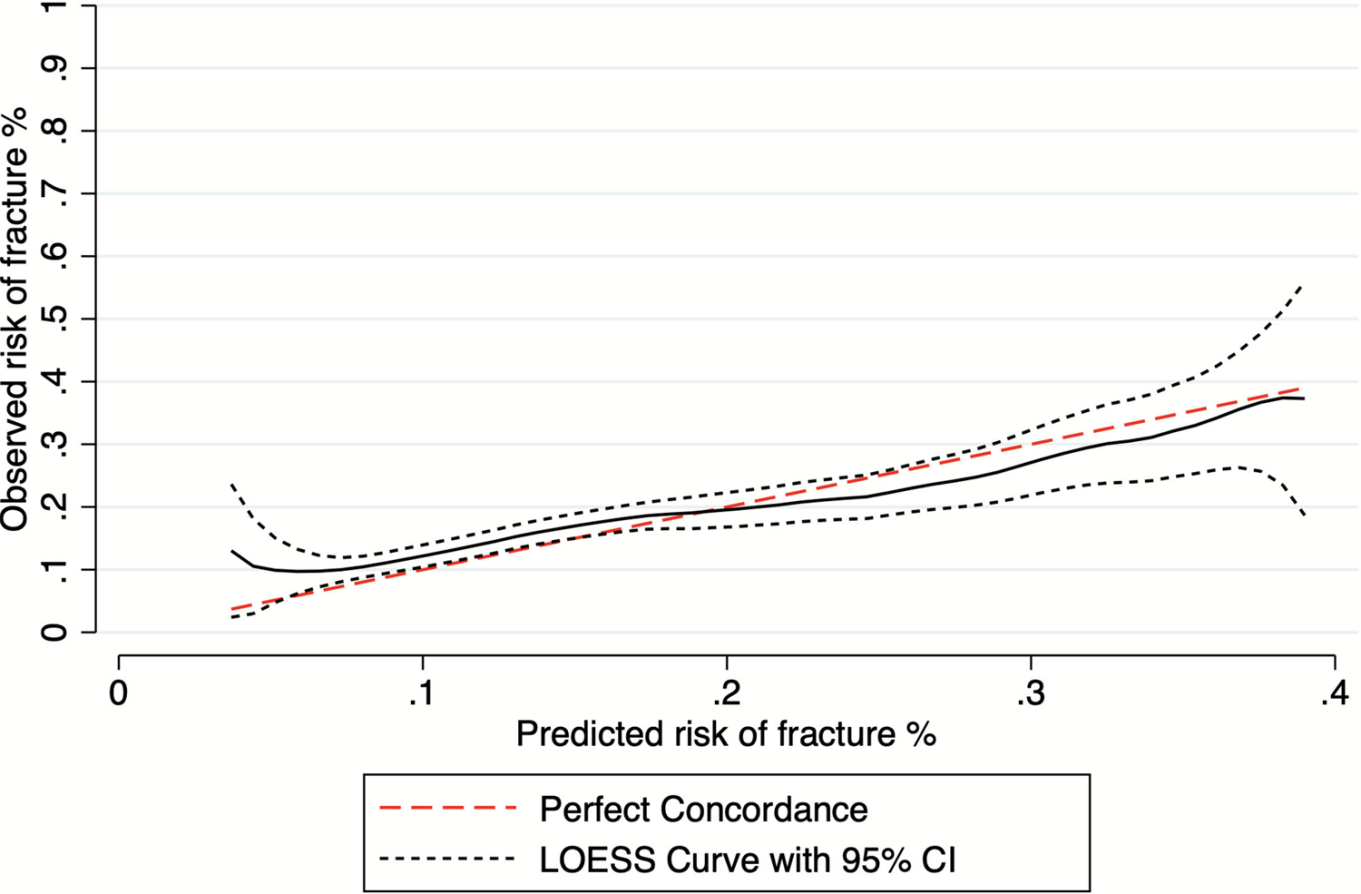
Concordance plot illustrating the predicted risk of fractures compared to the observed risk including all predictors used in FRAX, except neck aBMD

**Fig. 5:**
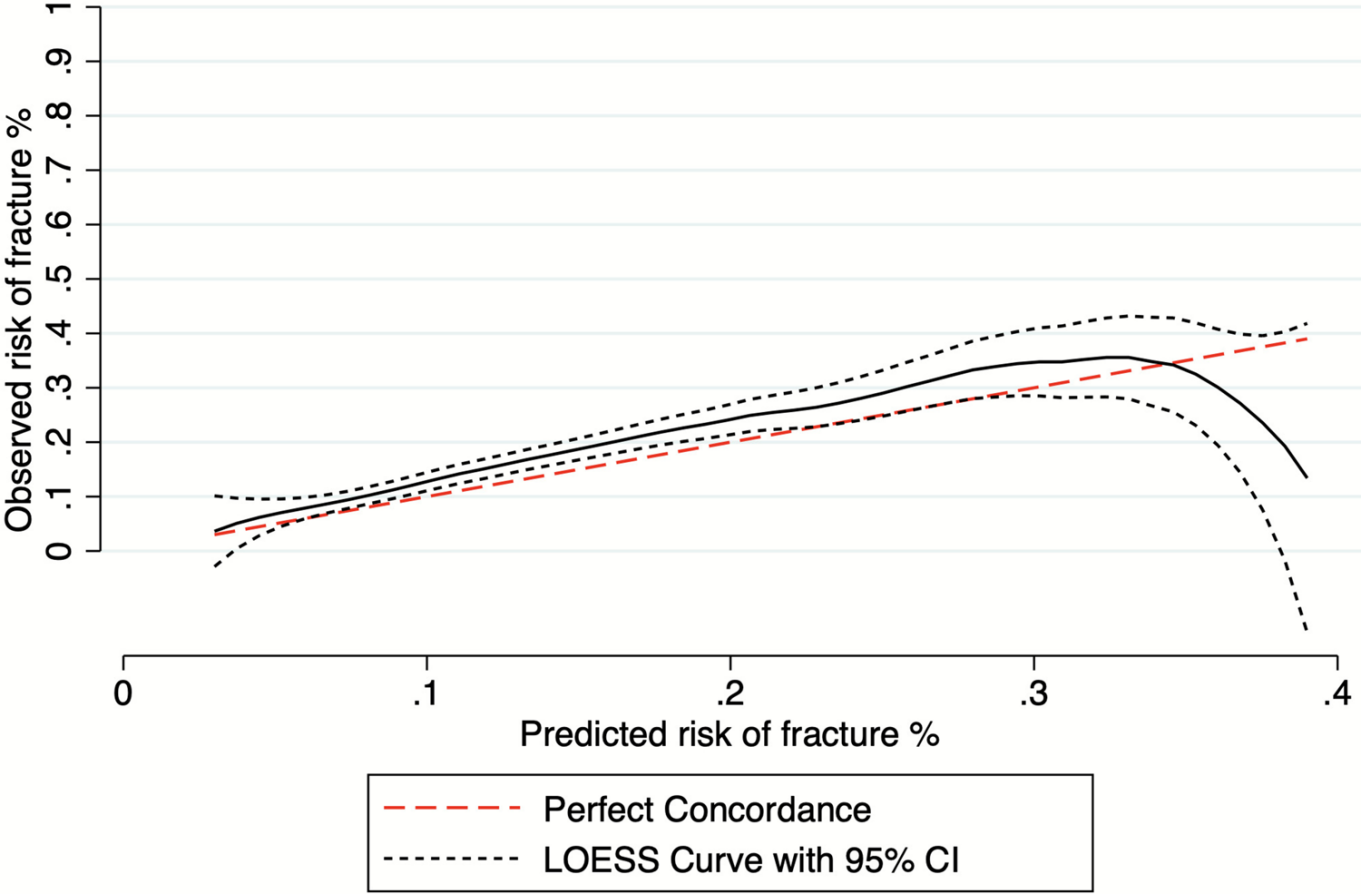
Concordance plot illustrating the predicted risk of fractures compared to the observed risk including all predictors used in FRAX, including neck aBMD

**Fig. 6:**
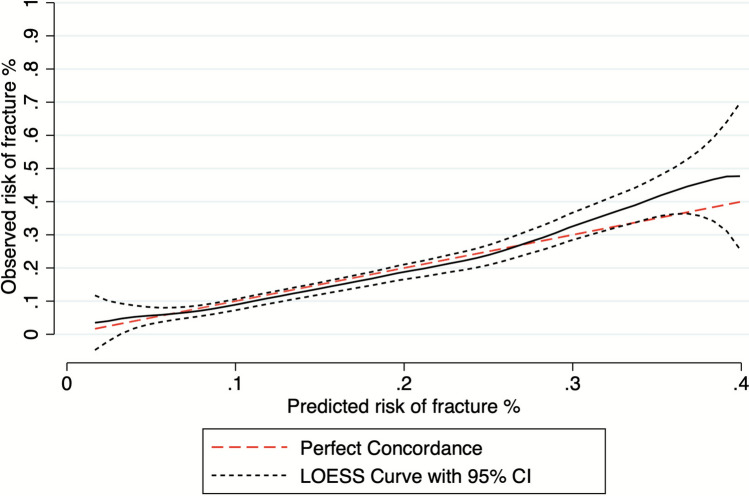
Concordance plot illustrating the predicted risk of fractures compared to the observed risk including all predictors used in FRAX, including neck aBMD and radius vBMD

Using the FRAX algorithm without BMD, the mean predicted 10-year fracture risk was 18.5% in those who fractured and 14.6% in the rest of the cohort, with a ROC-area of 62% (95% CI, 59–64%, Fig. 4). Adding femoral neck aBMD resulted in estimated risks of 16.7% and 12.6% in fractured and non-fractured individuals respectively, while the ROC-area increased to 66% (95% CI, 63–68%, Fig. 5). Concordance plots suggested a systematic underestimation of fracture risk when femoral neck aBMD was included. Adding radius vBMD did increase the risk estimates to 20.9% and 15.7% respectively, with a ROC-area of 68% (95% CI, 65–70%, Fig. 6).

### Determination of treatment thresholds

To determine the optimal threshold for initiating bone-specific treatment, the ROC-area was analyzed for different risk thresholds using SHAFRE and FRAX models (Tables 3, 4). Using SHAFRE, the ROC-area peaked at approximately 15% estimated fracture risk across all models, with the highest value of 64% in the model incorporating both aBMD and vBMD (Table 3). In FRAX, the optimal treatment threshold varied between 13 and 17%, depending on whether aBMD and vBMD were included in the model (Table 4).

**Table 3:** Validation of different thresholds for the predicted individual 10-year risk of fracture in the total population using SHAFRE and evaluating three different models

Threshold%	SHAFRE without bone density			SHAFRE with neck aBMD			SHAFRE with neck aBMD and radius vBMD		
	Sensitivity	Specificity	ROC-area	Sensitivity	Specificity	ROC-area	Sensitivity	Specificity	ROC-area
10	83.0	54.0	58.5	88.0	29.4	58.7	87.7	32.9	60.3
11	80.9	38.4	59.6	85.7	35.1	60.4	84.3	38.1	61.2
12	78.0	42.7	60.3	81.9	41.1	61.5	81.4	42.8	62.1
13	77.1	43.8	60.5	78.5	46.1	62.3	78.5	48.4	63.4
14	76.4	44.7	60.6	74.1	52.0	63.0	75.7	53.4	64.5
15	75.3	46.9	61.1	70.7	56.7	63.7	70.7	57.7	64.2
16	69.2	53.0	61.1	66.4	60.8	63.6	66.6	61.5	64.0
17	57.8	62.2	60.0	62.8	65.2	64.0	63.2	65.4	64.3
18	46.3	72.3	59.3	59.2	68.9	64.0	59.4	69.2	64.3
19	41.5	76.8	59.2	54.4	72.4	63.4	55.1	72.1	63.6
20	39.9	78.3	59.1	49.7	75.4	62.6	52.4	74.8	63.6
21	38.6	79.6	59.1	45.6	77.9	61.7	49.0	77.8	63.4
22	32.6	84.8	58.7	42.2	80.7	61.5	46.7	80.1	63.4
23	29.9	86.4	58.1	39.5	83.0	61.3	44.4	82.3	63.3
24	29.5	86.6	58.1	36.1	85.0	60.6	39.4	84.6	62.0
25	29.2	87.0	58.1	33.1	87.1	60.1	36.9	86.5	61.7

The first did included all SHAFRE predictors, the second included all SHAFRE predictors and neck aBMD, and the last included all SHAFRE predictors and neck aBMD, and radius vBMD. Specificity, sensitivity and the receiver operation characteristic area (ROC-area) are presented

**Table 4:** Validation of different thresholds for the predicted individual 10-year risk of fracture in the total population using FRAX and evaluating three different models

Threshold%	FRAX without neck aBMD			FRAX with neck aBMD			FRAX with neck aBMD and radius vBMD		
	Sensitivity%	Specificity%	ROC-area%	Sensitivity%	Specificity%	ROC-area%	Sensitivity%	Specificity%	ROC-area%
10	78.4	36.5	55.5	75.9	44.8	60.3	89.5	25.2	57.3
11	76.6	38.8	57.7	71.2	48.8	60.0	85.7	31.4	58.6
12	73.4	43.8	58.6	66.7	56.2	61.5	81.8	38.0	59.9
13	67.1	49.2	58.1	61.7	62.0	61.9	77.6	44.0	60.8
14	60.5	56.0	58.2	57.6	66.1	61.8	73.9	49.6	61.7
15	55.3	62.9	59.1	52.2	70.1	61.2	69.8	55.2	62.5
16	48.3	68.3	58.3	47.6	73.5	60.5	66.4	60.6	63.5
17	43.5	72.1	57.8	42.8	77.3	60.0	63.3	65.2	64.3
18	40.8	74.5	57.6	37.3	80.3	58.8	59.2	69.1	64.2
19	38.6	76.7	57.7	34.2	83.0	58.6	54.4	72.4	63.4
20	36.3	78.3	57.3	30.2	85.4	57.8	50.3	75.7	63.0
21	33.3	80.2	56.7	29.2	86.9	58.0	44.2	78.9	61.5
22	31.3	81.7	56.5	25.9	88.6	57.3	40.6	81.9	61.2
23	28.3	86.0	56.1	22.7	89.6	56.2	36.9	83.5	60.2
24	26.8	86.0	56.4	20.9	91.1	56.0	32.7	85.5	59.1
25	24.0	87.5	55.8	17.5	92.1	54.8	29.0	87.2	58.1

The first did included all FRAX predictors, except neck aBMD, the second included all FRAX predictors, and the last included all FRAX predictors and radius vBMD. Specificity, sensitivity and the receiver operation characteristic area (ROC-area) are presented

## Discussion

In this population-based cohort of older adults, both the SHAFRE and FRAX algorithms demonstrated only moderate ability to discriminate individuals who would later sustain a fracture, with ROC-areas ranging from 62 to 70% across models. Adding femoral neck aBMD improved discrimination for both algorithms compared with clinical risk factors alone, but FRAX still systematically underestimated absolute fracture risk in this cohort. Adding radial vBMD on top of aBMD did not materially improve predictive performance for either algorithm, as reflected by overlapping 95% confidence intervals. Notably, threshold-specific analysis identified an optimal predicted fracture risk of 13–17%, consistently lower than the commonly recommended 20% threshold for initiating bone-specific treatment in many clinical guidelines.

Using SHAFRE without BMD the estimated fracture risk was 20.4% in individuals suffering fractures during follow-up, compared to 15.9% in the rest of the cohort, with a suggested optimal threshold for initiation of bone-specific treatment of 15%. This threshold was associated with a ROC-area of no more than 64%, suggesting rather poor predictive ability. The ability to predict fractures improved only if both measures of bone density was added to the model. However, the ROC-area for the optimal treatment threshold still remained low at a maximum of 64%. Similar, and a little lower estimate of precision was found for FRAX, and in addition the FRAX model underestimated the risk of fractures if not both measures of bone density was added to the model. It should be noted that the addition of vBMD to FRAX and SHAFRE was exploratory. The overlapping 95% confidence intervals for the AUCs indicate that adding vBMD did not significantly improve the discriminatory ability of either model. In addition, the use of vBMD in the clinic is of limited value due to the high radiation dose and associated scanning times. In a recent study, we presented data for the SHAFRE model for the outcome of hip fractures in individuals aged 50 years and older, and also the optimal treatment threshold was analyzed. Thus, the ROC-area for hip fracture peaked at 77% in both men and women without adding any measures of bone density [9]. This higher accuracy has also been demonstrated for FRAX, [6] and has several explanations. These include a less heterogeneous group that is suffering hip fractures, and that hip fractures are captured in registers with greater precision than other fractures [15–18]. Irrespectively, the higher predictive ability suggests that the focus in the clinic should be on predicting hip fractures. In addition, a shift toward a focus on predicting hip fractures is also suggested by the high mortality after hip fracture, [21] and since a substantial proportion of hip fracture patients will lose independency in need of sheltered living [22].

Regardless, the threshold for initiating bone-specific treatment is of importance. According to most guidelines, the threshold for treatment with bone-specific agents is an estimated fracture risk of 20%. This threshold is most often based on consensus and expert opinion rather than on simulations from clinical data. Thus, a review that evaluated 120 guidelines for treatment thresholds that incorporated the FRAX-tool concluded that in almost all cases, there is no rationale given for the threshold chosen, [7] except that it is used by the National Osteoporosis Foundation of the USA [23]. This guideline suggests intervention thresholds of 3% for hip fractures and 20% 10-year risk of a major fracture as intervention thresholds. This guideline is based on a health economic analysis that examined the cost-effectiveness of pharmaceutical intervention [24]. A few other guidelines have instead suggested guidelines based on maximal discrimination, [25] or that the estimated fracture risk in those with fracture should be used as an intervention threshold [26]. Based on any of these definitions, the threshold for a major fracture of about 20% seems higher compared to the estimates in the present cohort based on a maximized AUC, with an accuracy that was less than desirable, as discussed above. Regardless, to guide clinicians in decision-making and simplify initiation of bone-specific treatment, it would be of importance for guidelines to be harmonized across different countries and regions.

While this study provides valuable insights, it is important to acknowledge its limitations. The study population was recruited from a specific geographic region. Thus, it would be of value if similar studies were performed in populations emanating from other regions and populations. Although parental hip fracture and self-reported alcohol intake were not available and replaced with registry-based measures (sibling hip fracture and diagnosed alcohol abuse), previous FRAX validation studies indicate that such lifestyle and family-history variables contribute only marginally to model discrimination, [27] and that parental and sibling histories of fracture carry the same significance for future fracture risk [28]. It is also clear that the number of fractures was limited, which may have influenced the statistical power. Yet, the population and number of fractures were larger than in most other studies including both vBMD and aBMD as exposures with the primary outcome of fractures [29–35]. The strengths include the population-based sample, measurements of both vBMD and aBMD, in addition to other known risk factors for fractures, and follow-up using nationwide register with virtual no loss at follow-up.

In conclusion, both SHAFRE and FRAX demonstrated similar and only modest ability to predict major osteoporotic fractures when evaluated head-to-head within the same cohort. This aligns with previous research indicating limited predictive accuracy for major fracture outcomes using FRAX. Our study extends the existing literature by providing the first direct comparison of the two algorithms using identical predictor information, including BMD and exploratory vBMD. Taken together, these findings highlight the need for greater clinical emphasis on hip fracture prediction, where both models have shown substantially stronger discriminative performance in larger cohorts.

## Data Availability

Restrictions apply to the availability of some, or all data generated or analyzed during this study to preserve patient confidentiality or because they were used under license. The corresponding author will on request detail the restrictions and any conditions under which access to some data may be provided.
